# Anti‐EGFR antibody‐drug conjugate for triple‐negative breast cancer therapy

**DOI:** 10.1002/elsc.202000027

**Published:** 2020-10-07

**Authors:** Yingnan Si, Yuanxin Xu, JiaShiung Guan, Kai Chen, Seulhee Kim, Eddy S. Yang, Lufang Zhou, Xiaoguang Margaret Liu

**Affiliations:** ^1^ Department of Biomedical Engineering University of Alabama at Birmingham (UAB) Birmingham AL USA; ^2^ Department of Medicine UAB Birmingham AL USA; ^3^ Department of Radiation Oncology O'Neal Comprehensive Cancer Center at UAB Birmingham AL USA

**Keywords:** antibody‐drug conjugate, epidermal growth factor receptor, targeted therapy, triple‐negative breast cancer

## Abstract

Triple‐negative breast cancers (TNBCs) are highly aggressive, metastatic and recurrent. Cytotoxic chemotherapies with limited clinical benefits and severe side effects are the standard therapeutic strategies, but, to date, there is no efficacious targeted therapy. Literature and our data showed that epidermal growth factor receptor (EGFR) is overexpressed on TNBC cell surface and is a promising oncological target. The objective of this study was to develop an antibody‐drug conjugate (ADC) to target EGFR^+^ TNBC and deliver high‐potency drug. First, we constructed an ADC by conjugating anti‐EGFR monoclonal antibody with mertansine which inhibits microtubule assembly via linker Sulfo‐SMCC. Second, we confirmed the TNBC‐targeting specificity of anti‐EGFR ADC by evaluating its surface binding and internalization in MDA‐MB‐468 cells and targeting to TNBC xenograft in subcutaneous mouse mode. The live‐cell and live‐animal imaging with confocal laser scanning microscopy and In Vivo Imaging System (IVIS) confirmed the TNBC‐targeting. Finally, both in vitro toxicity assay and in vivo anti‐cancer efficacy study in TNBC xenograft models showed that the constructed ADC significantly inhibited TNBC growth, and the pharmacokinetics study indicated its high circulation stability. This study indicated that the anti‐EGFR ADC has a great potential to against TNBC.

**Abbreviations**
ADC: antibody‐drug conjugate
DAR: drug‐antibody ratio
DM1: mertansine
EGFR: epidermal growth factor receptor
HER2: human epidermal growth factor receptor 2
PI3K: phosphorylating phosphatidylinositol 3‐kinase
TNBC: triple‐negative breast cancer

## 1 INTRODUCTION

Triple‐negative breast cancers (TNBCs), lacking the biomarkers of estrogen receptor, progesterone receptor, or human epidermal growth factor receptor 2 (HER2), account for about 15% of all breast cancers and are characterized by rapid growth, metastasis, and high recurrence [1]. Unfortunately, the severe adverse effects and drug resistance associated with standard cytotoxic chemotherapies (e.g. anthracylines, cyclophosphamide, and taxanes) minimize their clinical benefits in TNBC treatment [2, 3, 4]. There remains no efficacious targeted therapy for TNBC patients.

Epidermal growth factor receptor (EGFR) is a transmembrane receptor tyrosine kinase in plasma membrane, nuclear membrane, and other cellular components [5]. EGFR is overexpressed in various tumors, e.g. TNBC (52‐54%) [5, 6], lung cancer (40%) [7, 8], glioblastoma (50%), and head and neck cancers (80‐90%), while nearly undetectable in the corresponding normal organs [5, 6, 9]. Literature [5, 10‐12] showed that EGFR is a promising oncological target in both human [13, 14, 15] and mouse [16, 17, 18] TNBCs. Moreover, EGFR stimulates cell proliferation signaling via phosphorylating phosphatidylinositol 3‐kinase (PI3K) [19, 20] and enhances the DNA replication and repair through BRCA1 [21, 22, 23, 24]. Furthermore, monoclonal antibodies (mAbs) that target EGFR and folate receptor have been developed to treat TNBC, but the efficacy is limited as a single agent in clinical trials.

Antibody‐drug conjugate (ADC) is a targeted cancer therapy that combines the cancer specificity characteristic of antibody and the cytotoxicity of chemotherapy. Up to now, three ADCs, i.e. brentuximab vedotin, trastuzumab emtansine, and traztuzumab deruxtecan [25, 26, 27, 28, 29, 30, 31, 32, 33], have been approved by FDA to treat relapsed Hodgkin lymphoma and systemic anaplastic large cell lymphoma [34], relapsed or chemorefractory HER2‐positive BC [35], and previously treated or advanced HER2‐psotive BC [32, 33], respectively. Mertansine (DM1) is a cytotoxic agent synthesized to inhibit the assembly of microtubules during cell proliferation [36], which effectively treats cancer in the ADC trastuzumab emtansine. Compared to the chemotherapy or mAb‐based therapy, ADC can carry highly potent drugs that are too toxic to be used as chemotherapy, target the surface receptor overexpressed in cancer cells via mAb, specifically deliver drugs, and trigger antibody‐dependent cellular cytotoxicity or immune responses. Despite these promising achievements, no ADC therapy has been clinically used for TNBC treatment.

The objective of this study was to develop an ADC to treat EGFR‐overexpressing TNBCs. The ADC was developed by conjugating anti‐EGFR mAb with a potent cytotoxic payload DM1 that blocks microtubule polymerization. The TNBC‐specific targeting, plasma stability, and anti‐cancer efficacy were investigated using TNBC cell line and xenograft mouse model. Our results showed that the anti‐EGFR ADC is a promising targeted therapy for TNBC.

**PRACTICAL APPLICATION**

Due to the high malignance, metastasis and recurrence of TNBCs, the combined chemotherapies (main clinical option) and immunotherapy‐chemotherapy (i.e. Atezolizumab and Abraxane to treat PD‐L1^+^ TNBC) have more clinical advantages than single agent. This study developed and evaluated an ADC by integrating antibody with a cytotoxic small molecule, providing a new targeted therapy to treat EGFR^+^ TNBC. This therapy offers several anti‐cancer advantages such as cancer targeting, microtubule destruction, proliferation modulation, and antibody‐dependent cellular cytotoxicity. Moreover, the antibody and drug conjugation procedure is robust and scaleble for future biomanufacturing. The obtained results can facilitate the translation of this candidate therapy to future clinic, which will ultimately benefit patients with TNBC.

## 2 MATERIALS AND METHODS

The animal studies performed in this research conform to the National Institutes of Health (NIH) Guideline for the Care and Use of Laboratory Animals (Publication No. 85‐23). The animal protocol (IACUC‐21949) was approved by the Institutional Biosafety Committee at the University of Alabama at Birmingham.

### 2.1 Cell lines and media

The normal breast epithelium cell 184B5, HER2^+^ breast cancer cell BT474, and TNBC cell lines MDA‐MB‐468, MDA‐MB‐231 (ATCC, Manassas, VA), MDA‐MB‐468‐Luc and MDA‐MB‐231‐Luc (firefly luciferase, a bioluminescent reporter, GenTarget, San Diego, CA) were maintained in DMEM/SF12 supplemented with high glucose (4 g/L), l‐glutamine (4 mM), 10% fetal bovine serum (v/v), and penicillin (100 IU)‐streptomycin (100 μg/mL). Cell cultures in T‐flasks were incubated at 37°C and 5% CO_2_ in a humidified incubator (Caron, Marietta, OH). All media, supplements, chemical reagents, and assay kits were purchased from Thermo Fisher Scientific (Waltham, MA), unless otherwise specified.

### 2.2 ADC conjugation, purification, characterization and integrity analysis

**FIGURE 1:**
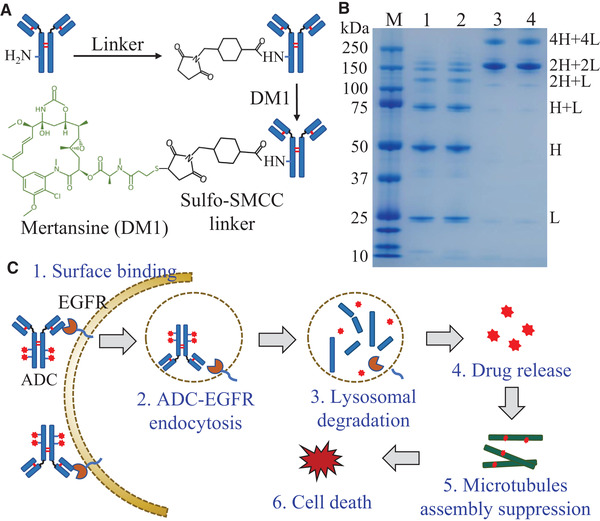
ADC construction, characterization and anti‐cancer mechanism. (A) The diagram of ADC construction: mAb was modified by the cross linker Sulfo‐SMCC via lysine, then DM1 was conjugated with mAb via linker to form ADC. (B) ADC was purified using HPLC and the structure integrity was confirmed with SDS‐PAGE. M: marker; 1 and 2: denatured anti‐EGFR ADC; 3 and 4: integral anti‐EGFR ADC. (C) Mechanism of ADC to treat TNBC: 1. ADC targets and binds to the surface receptor EGFR to form ADC/EGFR complex; 2. ADC internalizes and endocytoses; 3. ADC is degraded by lysosome; 4. Drug DM1 is released; 5. DM1 inhibits the assembly of microtubule by binding to tubulin; 6. TNBC inhibits cell proliferation and programs cell death

The ADC was constructed following our previously established conjugation procedure [36]. As shown in Figure 1A, 10 mg/mL anti‐EGFR mAb and 22.9 mM Sulfo‐SMCC linker (sulfosuccinimidyl 4‐[*N*‐maleimidomethyl] cyclohexane‐1‐carboxylate) were mixed with molar ratio of 1:10, incubated for 30 min at room temperature, and purified with 10 kDa MWCO concentrator. Then, the purified mAb‐linker complex reacted to DM1 with molar ratio of 1:20 for 30 min at room temperature. The ADC product was purified using HPLC (Shimadzu, Columbia, MD); the drug‐antibody ratio (DAR) was analyzed using HPLC UV absorbance spectra following our published method [36]; and the integrity of ADC was confirmed with SDS‐PAGE (Figure 1B). The NGC system was equilibrated with buffer A comprised of 0.02 M sodium phosphate and 0.02 M sodium citrate at pH 7.5 and eluted with buffer B contained 0.02 M sodium citrate and 0.1 M sodium chloride at pH 3.0. The purified ADC was neutralized to pH 7.0 with 1 M Tris solution, sterilized, and mixed with 0.1% sodium azide for long‐term storage at –80°C.

### 2.3 Flow cytometry

The overexpression of surface EGFR in TNBC MDA‐MB‐468 was confirmed with BD LSRII Flow cytometer (BD Biosciences, San Jose, CA). The 184B5 and BT474 were used as control cells. Cells were stained with 1 μg anti‐EGFR mAb‐AF647 (Alexa Fluor 647) per million cells for 30 min at room temperature in the dark and washed with PBS before analysis.

### 2.4 TNBC‐specific targeting and biodistribution

#### 2.4.1 Live‐cell confocal imaging

The surface binding and internalization of anti‐EGFR ADC in TNBC cells was evaluated using confocal microscopy following our reported protocol [36, 37, 38]. Briefly, the MDA‐MB‐468 cells were infected with BacMam GFP transduction control (BacMam 2.0) for plasma and nucleus staining for 24 h and observed using an Olympus 1X‐81 confocal microscope with an Olympus FV‐1000 laser scan head (Olympus IX81, Center Valley, PA). The ADC‐AF647 was then mixed with cells to collect the dynamic profiles of surface binding and internalization. The confocal images were processed using software FV10‐ASW 4.2 Viewer.

#### 2.4.2 Xenograft model and In Vivo Imaging System (IVIS) imaging

TNBC xenograft mouse model was generated by subcutaneously injecting 1 × 10^6^ MDA‐MB‐468‐Luc cells mixed with 25 μL matrigel into the mammary fat pad of 6‐wk‐old NSG (NOD scid gamma) female mice purchased from Jackson Labs (Bar Harbor, ME). About 2 wk post‐xenograft, the mice bearing ∼100 mm^3^ solid tumor were selected for ADC bio‐distribution and TNBC‐specific targeting analysis. The anti‐EGFR ADC was labeled with Cyanine 5.5 (Lumiprobe, Hunt Valley, MD) according to the manufacturer's protocol. The ADC‐Cy5.5 was injected into xenograft mice via tail vein with dose of 30 μg/mouse. Mice were imaged at 24 h post‐injection under IVIS Lumina Series III (PerkinElmer, Waltham, MA) with wavelength of 660 nm/710 nm (excitation/emission) and exposure time of 10 s.

### 2.5 Pharmacokinetics (PK)

The circulation stability of constructed anti‐EGFR ADC was investigated by intravenously (i.v.) administering four doses of ADC, i.e. 4, 8, 12, and 16 mg/kg‐body weight [BW], into 8‐wk NSG (NOD scid gamma) female mice purchased from Jackson Labs (Bar Harbor, ME). The 10‐μL blood samples were collected from tails at 2, 8, 24, 48, 72, and 120 h post‐injection and centrifuged at 2000 g for 5 min to collect supernatant for ELISA titration. The previously developed PK model was used to calculate half life *t*_1/2_ = 0.693*V*_d_/CL = ln(2)/k_e_ = 0.693/k_e_, recommended dose *D* = *C*_max(desired)_ *k*_e_⋅*V*_d_⋅*T*⋅(1‐e^−keτ^)/(1‐e^−keT^), and dosing interval *τ* = ln(*C*_max(desired)_/*C*_min(desired)_)/*k*_e_ + T. The calculated *D* and *τ* were used in the following in vivo anti‐cancer efficacy animal study.

### 2.6 Anti‐TNBC efficacy

#### 2.6.1 In vitro cytotoxicity assay

The MDA‐MB‐468 cells were seeded in 96‐well plates with viable cell density of 1 × 10^5^ cells/mL and viability of >95% and incubated for 24 h in CO_2_ incubator. The sterilized ADC was added to treat TNBC cells with final concentrations of 0, 50, and 100 nM. The cell viability was measured using Luminescent Cell Viability Assay (Promega, Madison, MI) at 72 h post‐treatment [36].

#### 2.6.2 In vivo anti‐cancer efficacy

The TNBC MDA‐MB‐468‐Luc xenograft NSG (NOD scid gamma) female mice were randomized into 2 groups (*n* = 5). The saline (control) or anti‐EGFR ADC was administrated intravenously through tail vein. Four injections were conducted on days 2, 6, 9, and 13 following a dose of 4 mg/kg‐BW in 50 μL. In addition to MDA‐MB‐468‐Luc (EGFR^+++^, high level of EGFR expression) xenograft model, we also generated MDA‐MB‐231‐Luc (EGFR^+^, medium level of EGFR expression) xenograft model to test the anti‐cancer efficacy of the constructed anti‐EGFR ADC. MDA‐MB‐231‐Luc xenograft NSG female mice were randomized into 3 groups (*n* = 5). Phosphate buffered saline (PBS), mAb and ADC (12 mg/kg‐BW) was i.v. injected into different mice groups on day 3, 6, 8, and 10. Tumor size was measured using an electronic caliper.

## 3 RESULTS AND DISCUSSION

In this study, we constructed and evaluated an EGFR‐targeting ADC for TNBC treatment. In ADC conjugation, the potent chemical drug, DM1 that induces apoptosis by blocking the assembly of microtubule, was reacted with mAb via linker (Figure 1A). The Sulfo‐SMCC linker reacted with the 10 lysine residues of anti‐EGFR mAb. We calculated the DAR following the published method [36]:

$$\mathrm{DAR}=\frac{\varepsilon_{\mathrm{mAb}}^{252}{-R\varepsilon }_{\mathrm{mAb}}^{280}}{{R\varepsilon }_{\mathrm{Drug}}^{280}{-\varepsilon }_{\mathrm{Drug}}^{252}},$$

where *R* = A_252_/A_280 _= Absorbance ratio, *ε*_mAb_^252 ^= 9.41 × 10^4^ M^−1^ cm^−1^, *ε*_mAb_^280 ^= 2.34 × 10^5^ M^−1^ cm^−1^, *ε*_DM1_^252 ^= 2.64 × 10^5^ M^−1^ cm^−1^, *ε*_DM1_^280 ^= 5.23 × 10^3^ M^−1^ cm^−1^. The DAR of the anti‐EGFR ADC constructed in this study was 1.6 and SDS‐PAGE confirmed the structure integrity of ADC (Figure 1B). The mechanism of cancer treatment with the ADC‐delivered drug was described in Figure 1C. First, the circulated ADC targets surface receptor EGFR in TNBC cells and forms ADC‐EGFR complex which could inhibit the phosphorylation of PI3K, block its signaling pathway, and downregulate the proliferation of TNBC cells. Second, the complex is internalized through receptor‐mediated endocytosis and localizes in cytoplasm. The intracellular ADC can bind to nuclear EGFR and inhibit DNA repair, which plays an important role in cancer treatment when combined with a synthetic lethal chemotherapy such as carboplatin. Third, lysosome degrades mAb and releases the cytotoxic DM1 as free drug. Finally, DM1 binds to tubulin and induces apoptosis and programmed cell death.

**FIGURE 2:**
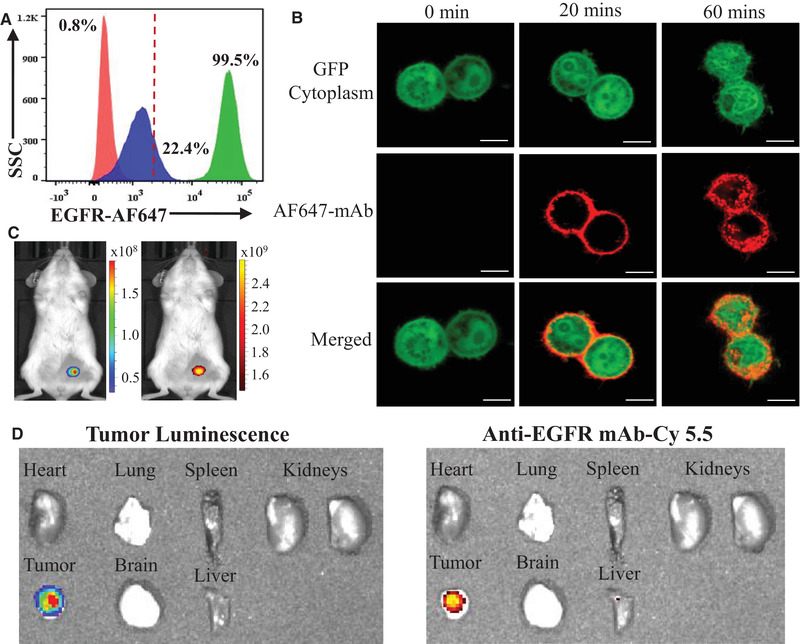
Evaluation of EGFR overexpression and ADC specific targeting to TNBC. (A) Surface expression assessment of EGFR in normal cell 184B5 (red), HER2^+^ cell BT474 (blue), and MDA‐MB‐468 (green) by flow cytometry. (B) Live‐cell dynamic imaging of surface binding and internalization of ADC in MDA‐MB‐468 cells by confocal laser scanning microscopy. Cells were stained with 1 μg of ADC‐AF647/million cells at 37°C and images were collected at 0, 20, and 60 min. Scale bar equals to 10 μm. (C) In vivo evaluation of specific targeting to TNBC (MDA‐MB‐468‐Luc) xenograft by IVIS. Total of 30 μg ADC‐Cy5.5 was intravenously injected into mice (*n* = 2) through tail vein. Live‐animal images were taken at 24 h post ADC injection. (D) The ex vivo analysis of the tumor and important organs (heart, lung, spleen, kidney, brain and liver)

In order to evaluate the anti‐EGFR ADC, we first performed flow cytometry to confirm EGFR surface expression in TNBC cells (Figure 2A). The normal cell 184B5 (control), HER2^+^ BT474 (control), and TNBC MDA‐MB‐468 showed EGFR expression rate of 0.8, 22.4, and 99.5%, respectively. This result is consistent with the clinical data showing that EGFR is overexpressed in most human TNBC samples [5, 6]. The minimal or low expression of EGFR in normal cells and HER2^+^ cells also indicated that EGFR is a good target candidate for TNBC. Then, we assessed the TNBC‐specificity of anti‐EGFR ADC both in vitro and in vivo. Specifically, live‐cell confocal microscopy imaging was used to collect the dynamic profiles of ADC‐TNBC interaction as presented in Figure 2B. After mixing the ADC‐AF647 (displayed as red color) with EGFR^+^ MDA‐MB‐468 (green color), ADC targeted and bound to cell surface within 20 min and internalized into cytoplasm through endocytosis within 60 min. Furthermore, we evaluated the in vivo TNBC specificity and biodistribution of anti‐EGFR ADC using a TNBC (MDA‐MB‐468‐Luc) xenograft mouse model (Figure 2C). The live‐animal IVIS imaging showed that ADC targeted and accumulated in TNBC xenograft within 24 h post ADC‐Cy5.5 injection, as indicated by the overlap of bioluminescent signal (Luc) and fluorescent signal (Cy5.5). Moreover, we did not observe non‐specific targeting (indicated as fluorescent signal) in normal organs or tissues of mouse model such as heart, lung, spleen, kidney, brain and liver, as confirmed with the ex vivo IVIS imaging (Figure 2D). Altogether, these imaging studies demonstrated that anti‐EGFR ADC can specifically target TNBC and effectively deliver the conjugated cargos (cyanine‐5.5 or DM1).

**FIGURE 3:**
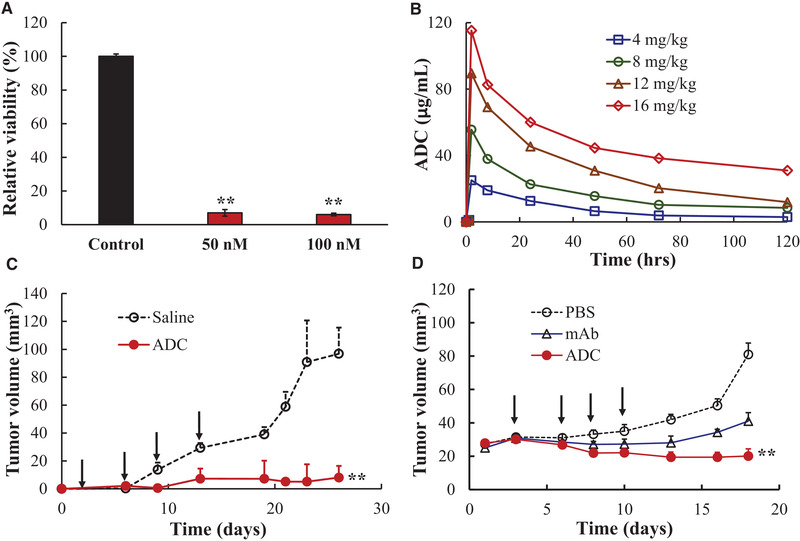
Evaluation of anti‐TNBC efficacy and pharmacokinetics of anti‐EGFR ADC. (A) In vitro anti‐TNBC cytotoxicity in MDA‐MB‐468 using Luminescent Cell Viability Assay. Data represent mean ± SEM, *n* = 3. (B) Pharmacokinetics study to evaluate the plasma stability and kinetics parameters of ADC (data represent mean ± SEM). (C) In vivo anti‐cancer efficacy study in TNBC (MDA‐MB‐468‐Luc) xenograft mouse model. Saline or ADC (4 mg/kg‐BW) was administrated on day 2, 6, 9, and 13 (arrow). (D) In vivo anti‐cancer efficacy study in TNBC (MDA‐MB‐231‐Luc) xenograft mouse model. Saline, mAb or ADC (12 mg/kg‐BW) were administrated on day 3, 6, 8, and 10 (arrow). Tumor size was measured with calipers and volume was calculated as ellipsoid. **P < 0.005 *vs*. control using ANOVA followed by Dunnett's *t*‐test. Data represent mean ± SEM, *n* = 5

Finally, we evaluated the TNBC treatment efficacy of ADC in vitro and in vivo. The in vitro anti‐cancer cytotoxicity assay was conducted on human TNBC MDA‐MB‐468 cells by testing two ADC doses, i.e. 50 nM and 100 nM. As illustrated in Figure 3A, MDA‐MB‐468 cells presented viability of 7% (50 nM) and viability of 6% (100 nM) compared to the control with viability of 100% (saline). Low viability indicated that the delivered DM1 had high potency to TNBC cells. Free (or unmodified) DM1 could be lethal to mice at low dose condition. The PK modeling (Figure 3B) showed that serum half‐life (t_1/2_) was 1.54‐2.83 days, recommended dose (D) was 5.78‐13.77 mg/kg, and recommended dosing interval (τ) was 4.04‐7.48 days. Guided with PK study, we selected a dose of 4 and 12 mg/kg of ADC with administration interval of 3 or 4 days, with anti‐EGFR mAb or saline as control, for the remaining in vivo anti‐TNBC study in female mice bearing TNBC xenografts (n = 5). Figure 3C showed that tumor growth was significantly attenuated with a 91.7% reduction of tumor volume in ADC treatment group compared to that in control groups (*P* ≤ 0.005), indicating that DM1 was successfully delivered to TNBC MDA‐MB‐468‐Luc xenograft and inhibited tumor growth. We also evaluated the anti‐tumor efficacy of anti‐EGFR ADC, with mAb and saline as controls, in TNBC MDA‐MB‐231‐Luc xenograft model. The results showed that ADC inhibited (or stopped) tumor growth (Figure 3D) while saline group had faster tumor growth than mAb and ADC control groups. The body weight was monitored but there was no obvious difference between the treatment group and control groups (data not shown), indicating there was no systemic toxicity of ADC treatment.

The quick and specific targeting (as shown Figure 2C) could reduce the side effects of the delivered cytotoxic DM1. These results demonstrated that anti‐EGFR ADC is an effective targeted therapy against TNBC. In addition to the FDA approved Trastuzumab emtansine [39], several DM1‐based ADCs, bivatuzumab mertansine [40], cantuzumab mertansine [41] and lorvotuzumab mertansine [42], are under clinical trial evaluation for the treatment of head, neck and lung cancers. Although the standard 3‐wk ADC treatment was performed to simulate the clinical ADC therapy, it is highly valuable to test the survival of treated mice, which will be performed in our future study.

## 4 CONCLUSION AND FUTURE OUTLOOK

In conclusion, ADC has more advantages or therapeutic values than antibody only or chemotherapy only for TNBC because it can target TNBC but not normal tissues, reduce undesirable side effects, and deliver small molecule that is too toxic to be used as therapeutic agent. In addition, the anti‐EGFR mAb in ADC can regulate tumor cell proliferation and inhibit DNA repair via modulating cell membrane or nucleus membrane EGFR. In future, we will further evaluate the integrated anti‐cancer mechanisms using immunocompetent model or humanized model. The combination with other therapies will also be investigated. We expect to improve the life quality and survival rate of patients with TNBC in future.

## CONFLICT OF INTEREST

The author Dr. Eddy S. Yang has the following conflicts of interest to disclose: Advisory board of Astrazeneca, Bayer, Clovis, and Strata Oncology, and Consultant of Eli Lilly. Other authors have declared no conflict of interest.
